# ‘This is like tradition, lie on your back, hold your leg, and push’: understanding midwives’ perspectives on their choice of labour positions in a Ugandan hospital

**DOI:** 10.1186/s12884-025-07657-2

**Published:** 2025-05-13

**Authors:** Zuhaira Husna Fatma, Helle Mölsted Alvesson, Gertrude Namazzi, Josephine Babirye Kyobe, Elizabeth Ayebare

**Affiliations:** 1 Department of Global Public Health, Karolinska Institutet, Stockholm, Sweden; 2 Department of Health Policy Planning and Management, School of Public Health, Makerere University, Kampala, Uganda; 3 Department of Nursing, School of Health Sciences, Makerere University, Kampala, Uganda

**Keywords:** Labour positions, Midwifery, Hospital setting, Respectful maternity care

## Abstract

**Introduction:**

Despite the well-documented benefits of upright positions and mobility during labour and childbirth, the adoption of these remains limited. Offering women choices in labour positions is essential for respectful maternity care. Since midwives play a pivotal role in guiding women through labour, their perspectives are crucial to the effective integration of these practices.

**Aim:**

The study aimed to understand midwives’ perspectives and reasoning behind their choice of labour positions.

**Methods:**

This qualitative study was conducted in a regional referral hospital in eastern Uganda. Twelve midwives were recruited purposively at two different time points in December 2020 and February 2024. A data-driven reflexive thematic analysis was conducted.

**Results:**

Midwives’ choice of labour positions was based on their understanding of the advantages and disadvantages for mothers and babies. Midwives were only open to changing their practices when they were confident in their knowledge and skills. The availability of resources influenced their perspectives on which positions were most feasible and effective in different situations. The midwives prioritised assessment of the mother’s clinical condition rather than her preferences when choosing birth positions.

**Conclusion:**

This study highlighted midwives’ willingness to support different and, to them, new labour positions when confident in their efficacy and safety. Addressing misconceptions about risks and equipment needs is therefore crucial. The prevailing provider-centric norms in hospitals may shape midwives’ approach to care, highlighting the need for supportive environments to foster midwives’ confidence in new practices.

**Supplementary Information:**

The online version contains supplementary material available at 10.1186/s12884-025-07657-2.

## Background

Throughout history, women have embraced a variety of labour positions, freely adjusting their posture based on personal preferences [1, 2]. However, the dominance of supine positions has emerged with the shift from home to institutional birth [2]. Nowadays, despite the World Health Organization (WHO) recommendation of various upright and ambulant positions—such as sitting, kneeling, squatting, sitting on a birthing stool, and walking—for low-risk women during the first stage of labour [3, 4], the practice of bed rest remains prevalent in many settings [3]. Similarly, during the second stage of labour, the WHO recommends that maternal health providers help women adopt their preferred birthing positions, including upright positions [5]. However, prevailing practices, particularly in low-income countries, often involve women lying on their backs [6, 7].

Numerous research studies have highlighted the advantages of mobility and upright positions for maternal and fetal outcomes [1, 8, 9]. During the first stage of labour, these have been associated with faster labour progression, a reduced likelihood of cesarean section, and a decreased need for neonatal intensive care [8]. Being upright has also been linked to a reduction in the duration of the second stage of labour, a decrease in assisted deliveries and episiotomies, and a lower occurrence of abnormal fetal heart rate patterns [1]. Moreover, it has been found to provide women with an enhanced sense of control and is perceived as empowering and less painful than the conventional supine posture [5, 9]. Nonetheless, in low-income settings, women’s perception of not being able to assume their preferred birthing positions may serve as a barrier to seeking care in healthcare facilities [10].

In Uganda, improving maternal health services to align with women’s birthing preferences is key to sustaining the progress in facility-based deliveries. Facility-based delivery is important, as it has been shown to reduce neonatal mortality by 29% in low- and middle-income countries [11]. In 2016, healthcare facility deliveries accounted for 73% of all live births in Uganda, which rose to 91% by 2022 [12]. Sustaining this progress in Uganda lies in fostering a culture of respectful maternity care, which includes respecting women’s preferences for labour positions [5, 13]. The Ministry of Health of Uganda recommends maternal health providers to encourage women to be mobile during the first stage of labour [14] and support women’s preferred position during all stages of labour [14, 15]. A cross-sectional study found that 84% of Ugandan women who gave birth at home preferred to give birth in other positions than lying on the back [13]. However, a 2020 study showed Ugandan women described feeling constrained by the labour practice in health facilities, which prevented them from experiencing childbirth in a manner aligned with their cultural norms and personal preferences [16]. In other studies, Ugandan women have expressed a desire for alternative labour positions, such as squatting or kneeling, which they feel limit the exposure of their genitalia during birth and lead to greater control and comfort [17, 18]. Furthermore, maternal mobility, a valued cultural tradition among some Ugandan women, is perceived as more attainable within the home environment [18]. These findings stress the importance of addressing women’s birthing preferences within maternal health services in Uganda to promote their utilisation.

## Theoretical framework

The midwifery model of childbirth highlights a holistic approach that focuses on women-centred care [19]. Midwifery care includes the provision of skilled, knowledgeable, and compassionate care for women, newborns, and families throughout all stages of pregnancy [20]. It involves optimising normal biological, psychological, social, and cultural processes, preventing and managing complications, collaborating with other healthcare services, respecting women’s choices and beliefs, and partnering with women to care for themselves and their families [20]. With the philosophy and care model of the midwifery practice, decision-making that involves pregnant women is important.

Foucault’s concept of normalisation underscores the pressure within institutional settings, like hospitals, to conform to established norms [21, 22]. This pressure is particularly evident in maternal health decisions, including those related to childbirth processes. Healthcare providers, including midwives, navigate a complex interplay of medical authority, institutional policies, and individual agency, shaping patient care accordingly [21, 22]. Institutional protocols, guidelines, and routines are instrumental in establishing a standard way of conducting procedures and influencing the behaviours and practices of professionals [21, 22]. According to Foucault, knowledge production within institutional contexts, such as hospitals, reinforces these norms, positioning institutions as authoritative determinants of normality and deviance [23]. Hence, it is relevant to consider institutional norms while exploring midwives’ perspectives and practice in hospital settings.

While existing research has focused on women’s preferences for labour positions [9, 24–26], there is limited knowledge about the factors influencing midwives’ decisions [24, 27, 28]. This study aimed to understand midwives’ perspectives and reasoning behind their use of various labour positions, which could guide interventions to help midwives deliver respectful, women-centred care. We utilised the midwifery model of care and Foucault’s theory as a lens to understand and discuss the results of the analysis.

## Methods

### Research design

This study is a qualitative descriptive design, using reflexive thematic analysis of semi-structured interviews with midwives, and was nested within the ALERT (Action Leveraging Evidence to Reduce Perinatal Mortality and Morbidity) project in Uganda [29]. We conducted two rounds of interviews with midwives. The first round was part of the ALERT formative assessment, aiming to inform an intervention to promote respectful maternity care and improve perinatal health. The second round focused specifically on labour positions and their connection to respectful care. This paper utilised the Consolidated Criteria for Reporting Qualitative Research (COREQ) checklist [30] to ensure transparency and credibility of the study procedures.

### Study settings

#### Study hospital

The study took place in the maternity unit of a public regional referral hospital in Uganda, where 5,000 deliveries occurred from July 2022 to March 2023, with 460 to 560 births monthly. The maternity ward staff included midwives, obstetricians, medical officers, interns, and students. The maternity ward is under the Department of Obstetrics and Gynaecology, headed by a consultant obstetrician/gynaecologist. The person in charge of the maternity ward is a midwife who takes on both clinical and administrative roles. Midwives were primarily responsible for labour care and uncomplicated vaginal births. During the first data collection, 18 midwives worked in the ward, and during the second, there were 15. They worked in three shifts, with two midwives per shift.

In this hospital, midwives generally encouraged mothers to move freely around the labour ward during the first stage of labour. However, by the second stage, it was common for women to deliver on their backs while lying on a bed.

#### The ALERT project in Uganda

The ALERT intervention focused on optimising care delivery in areas such as admissions, labour monitoring, immediate maternal and newborn care, and managing complications [29]. It prioritised competency-based training for midwives [29], with modules on respectful maternity care and promoting maternal mobility and upright birthing positions, highlighting the importance of allowing women to choose their birthing positions.

### Recruitment and participants

Midwives working in the maternity ward during the interview periods were purposively recruited. Eligible participants were professionally trained midwives, with varying educational levels and years of experience. Midwives were excluded if they were on sick or study leave or otherwise unavailable for interviews, or if they were interns or students. All recruitment was done face-to-face. The overall sampling for this research was based on Malterud’s information power [31]. The adequacy of the number of participants was assessed by considering the narrow study focus, variations in dialogue quality across interviews, the application of theory, and case analysis. No midwives refused to participate in this study.

Different midwives were interviewed in 2020 and 2024, with five participating in 2020 and seven in 2024. Four of the twelve midwives had received ALERT training on mobility and upright positions, with one also having receiving additional practical training on upright positions and birthing stools through another project. Participant characteristics are shown in Table 1.

**Table 1:** Participants characteristics

Characteristics	*N* = 12
Age (years)	
Less than 30	1
30–40	4
40–50	3
More than 50	3
Missing data	1
Length of work (years)	
Less than 10	3
10–15	5
More than 15	4
Level of education	
Certificate	2
Diploma	6
Bachelor	4

### Data collection

The first set of semi-structured interviews were conducted in December 2020. The interviews covered questions about experiences with intrapartum care delivery and the labour process, and facilitators in childbirth care provision. The interview guide included open-ended questions about the use of birthing position and mobility during labour. All interviews were conducted face-to-face and audio recorded.

The second set of interviews were conducted in February 2024 to gain deeper insights into midwives’ perspectives on the use of labour positions. An interview guide was developed based on the results of the first set of interviews and a new literature review. The interview questions covered experiences during the first and second stages of labour, labour positioning practices, perspectives about various birthing positions, and reasoning behind the use of different positions. Questions about birthing stools were added, due to their availability in the hospital. Continuous refinement of the questions and prompts to make them more understandable to the midwives was done throughout the interview process. A piece of paper with the interview questions was given to the participants during the interview to aid the clarity of the questions. Field notes from observation during the interview were taken to record non-verbal interactions and the setting of the labour ward. The interview questions can be found in Supplementary File 1.

Interviews were conducted in English. All participants were fluent in in both writing and speaking English as this is the formal language in Uganda which is used in all educational institutions. To make it convenient for the participants, both sets of interviews were conducted in a private room in the maternity ward. The interviews were done after the end of each shift to minimise interruption. However, the interviews were sometimes interrupted when the midwives were needed elsewhere. After the midwife returned, the last conversation was reiterated, and privacy and comfort were assessed and re-established.

### Data analysis

Reflective thematic analysis, as described by Braun and Clarke, was applied to the data [32]. All data from the two time points was analysed as one to gain a comprehensive understanding of the phenomenon. For the 2020 data, the initial step involved the researcher familiarising herself with the dataset and selecting interview portions relevant to the research question. For the 2024 data, initial coding was conducted concurrently with transcription. Repeated examination of all transcripts was done to grasp the overall essence of the data. ZHF undertook the coding whilst holding regular discussions with EA and HMA. Semantic and latent codes were generated through an inductive process, with continual comparisons with previously coded data units. After initial coding, further read-throughs were conducted to develop a shared meaning-based pattern. Codes were then clustered into sub-themes. Discussions between ZHF, EA and HMA were then held to determine the final themes. Coding for this analysis was done using NVIVO software (release 14.23.2) in a Mac operating system.

### Research team and reflexivity

The two interview rounds were conducted by different researchers. The 2020 interviews were carried out by four Ugandan female researchers; two midwives (EA and JKB), a nurse with a Master’s in Public Health (SN), and a doctor (GN). All of these researchers had qualitative research experience and no employment connection to the study site. One interviewer had prior contact with some of the participating midwives from a previous study.

In 2024, the second set of interviews was conducted by ZHF, an Indonesian medical doctor and public health master’s student with over three years of clinical experience, including work in maternity wards. Although fluent in English, it is not her first language. She had no prior contact with the facility or midwives before the study. ZHF spent a month in Uganda, including a week in the maternity ward, to build rapport with the midwives and get acquainted with the setting.

## Results

Three themes and seven sub-themes were developed from the thematic analysis of midwives’ accounts of various labour positions and these are displayed in Table 2 below.

**Table 2:** Themes and sub-themes

Overarching theme: Promoting childbirth practices that are aligned with midwives’ roles and needs		
Theme		Sub-theme
1	Understanding the pros and cons of labour positions drives change	Concerns and uncertainty about new labour positions
		Advantages of mobility and upright position
		Building blocks of change
2	Labour ward organization is not designed for diverse needs	Adequate physical resources and workforce to support any positions
		Navigating new tools within current context
3	Labour positions in alignment with women's condition and midwives? duties	Conditional use of labour positions
		Controlled birthing process

### Theme 1. Understanding the pros and cons of labour positions drives change

Concerns and uncertainty about new positions were driving midwives’ decisions on labour positions. Midwives noted that upright positions and mobility were common during the first stage of labour, while women typically lay on their backs during the second stage. They described uncertainty about supporting non-supine positions in the second stage, expressing concerns about the practicality of squatting and birth complications.

*“If the mother is squatting*,* how are you receiving this baby*,* so it’s not traumatised if it falls down?”* (Midwife, 3 years of experience).

Midwives acknowledged the advantages of mobility and upright positions, especially during the first stage, which led them to encourage women to be upright. Additionally, when considering the second stage of labour, midwives recognised the potential for an upright position to facilitate delivery and were willing to recommend it to specific women based on individual needs and circumstances.

*“Ok*,* we encourage them to walk around*,* sit on the bed*,* once in a while lie on the left lateral position*,* yes. To aid the descent of the presenting part.”* (Midwife, 10 years of experience).

*“[Support] according to the parity. With primigravida*,* I prefer lithotomy because we can be able to support the perineum or do an episiotomy. But with grand multiparas*,* squatting.”* (Midwife, 30 years of experience).

Midwives expressed how knowledge could influence practice. A better understanding of a position was presented as a catalyst for adopting it. Also, midwives stated that their colleagues’ views could influence how they perceived the new knowledge. They expressed that when other midwives supported different positions, they would themselves be more comfortable assisting women in assuming those positions.

*“But again*,* delivery there it doesn’t come*,* but sitting on it [birthing stool] I think it is good. But pushing when sitting on it*,* I’ve not yet got that. But*,* if the other midwives’ opinion about something is positive*,* it would be very easy for me to adopt. Very easy for me to adopt.”* (Midwife, 23 years of experience).

### Theme 2. Labour ward organization is not designed for diverse needs

Midwives noted that some positions were practically unfeasible due to the lack of supportive environmental conditions, such as squatting or kneeling on the floor in a shared labour suite with dirty floors, which could predispose the mother to infections.

*“When I saw it [birthing stool]*,* I asked*,* ‘sister*,* what’s this for?’ She said*,* ‘it’s for the mother to deliver on.’ I was like*,* ‘how? Where?’ They were like*,* ‘They sit on when they are on the second stage and deliver the baby.’ I was like*,* ‘Where do we place it? Because our floor is all dirty.’ How do we do it? Do we put the plastic sheet on the floor*,* then put the stool on*,* and she pushes*,* and the baby falls? And I think it’s too short*,* cause I have a problem with my back. How do I deliver? […]”* (Midwife, 23 years of experience).

Midwives also reported that staff shortages made their jobs challenging, leading them to prefer practices that reduce workload. The dorsal position was commonly chosen as it allows midwives to assist multiple deliveries consecutively. They also noted that sitting or squatting positions risked the baby falling if midwives were attending other deliveries.

*“Dorsal position*,* yes. And I think it’s also the most comfortable position for the midwives. Because now*,* like in this setting*,* […] you can find you are the only qualified midwife*,* and then you have like ten mothers in active labour or five. Then you find that they can push in 5 minutes. So*,* you just spread them on those beds*,* and when you see that head coming from there*,* just make sure you use many gloves*,* like three. Yes. When you see the other one is very fast coming*,* just take off one glove*,* just receive the baby*,* put on the mother’s abdomen*,* and cover the baby*,* then come and conduct another one.”* (Midwives, 10 years of experience).

The introduction of the birthing stool required midwives to adapt its use within their existing environment. Beds were still considered important in supporting labour positions, with midwives describing the practice of placing birthing stools on the beds to improve comfort for both mothers and themselves. Discussions on birthing stools mainly focused on their use during the first stage of labour, with midwives preferring to move women to lie down on beds as the second stage approaches.

*“[…] So*,* delivery bed—we need good ones which are firm*,* and we can make these mothers sit on those delivery beds on our delivery stool. They can also adopt any positions they need; as long as the delivery bed is comfortable*,* you can do everything.”* (Midwife, 15 years of experience).

### Theme 3. Labour positions in alignment with women’s condition and midwives’ duties

Midwives stated that the choice of labour positions usually depended on physiological cues and women’s characteristics. The physiological cues were seen as a time point to change from one position, mostly upright, to another, primarily lying on the back. Midwives stated that these decisions also stemmed from the concern for mothers’ well-being, and they believed that confining mothers in bed could prevent complications that may develop from being mobile or upright.

*“It’s [moving around] before the water has broken. Yeah. But as soon as the water breaks*,* you confine them in bed because we fear cord prolapse and infection*,* so we confine them in bed.*” (Midwife, 15 years of experience).

According to midwives, mother’s decisions were usually based on their previous birthing experience or lack thereof, as well as their physical capacity. Midwives would sometimes ask for the mothers’ preference, but they noted that women who had given birth multiple times were better able to position themselves.

*“We encourage them*,* which position they prefer*,* but squatting and kneeling is for grand multiparas.”* (Midwife, 30 years of experience).

Midwives acknowledged that women may not be fully aware of available positions or how to position themselves effectively. Regardless of parity, midwives always provided guidance during labour, as mothers may have forgotten instructions given during previous childbirth experiences.

*“If she’s in the second stage*,* I still teach her what to do. These mothers*,* even if she is delivering her fourth baby. They did forget what happens in labour suit.”* (Midwife, 2 years of experience).

Midwives felt a sense of responsibility to influence mothers’ decisions or behaviours regarding labour positions. They often guided mothers to close their legs or assume specific positions to ensure the safety of both mother and baby. Midwives mostly wished to uphold the labour practices they were accustomed to following, such as supporting the perineum and putting the baby on the mother’s body after birth. This led to hesitation in encouraging positions that prevented them from performing those practices. Midwives also noted that the lithotomy position as the prevailing practice was done as if it were “like a tradition”, and as a result, women were asked about their position preferences only in a few circumstances.

*“The mother has no choice like*,* ‘Musawo [healthcare worker]*,* I want to push while squatting.’ No. When they come*,* you tell them*,* ‘Go lie on the bed*,* lie on your back.’ […] She can’t tell you*,* ‘Musawo*,* me*,* I want this position.’ Because*,* even the Musawo*,* I don’t know any other position. This is like tradition: lie on your back*,* hold your leg*,* and push. So*,* there’s nothing like deciding. I grew up knowing it is the delivery position.”* (Midwife, 23 years of experience).

## Discussion

Exploring midwives’ perspectives on various labour positions showed an interplay of influences shaping their reasoning. These ranged from concerns about perceived risks in certain positions to collective judgments of safety among fellow midwives. Midwives highlighted the challenges of providing support without adequate resources. Moreover, their professional duty to balancing considerations of maternal and infant well-being played a significant role in shaping their approach to labour positioning.

Midwives often choose the labour practices that they feel most confident in carrying out, considering benefits and risks. Studies in Tanzania and China found that midwives’ reluctance to assist with upright positions stemmed not only from concerns about risks but also from uncertainty and a lack of knowledge about how to effectively support women in these positions [24, 27]. Although the midwifery model of care typically emphasises women’s natural ability and minimal intervention [33], a lack of knowledge about alternative birthing positions can hinder midwives from fully recognising and supporting women’s natural ability to give birth in non-supine positions. Nevertheless, upright positions do pose some risks, such as increased blood loss and second-degree perineal trauma, while also showing that they can reduce the rates of episiotomy, instrumental vaginal births, and abnormal fetal heart rate patterns [1]. The WHO has also stated that the benefits and harms of upright and supine positions might not be clinically different, highlighting the importance of encouraging women to adopt any position they find comfortable [5]. Several studies have shown that additional training may be needed to increase midwives’ confidence. A systematic review indicated that simulation-based training could improve the confidence of medical professionals caring for obstetric care [34]. Another review showed that mentoring, accompanied by on-the-job training and refreshers, could help improve midwives’ confidence in delivering care [35]. Given these considerations, it is crucial to address midwives’ preconceptions about the risks associated with upright labour positions and offer practical training to increase their confidence when incorporating these.

Besides knowledge and skills, midwives also perceive human and physical resources to be necessary to support upright labour positions. A study in South Africa highlighted the challenges midwives faced due to shortages in human resources and the limitations of the birthing environment, which hinder their ability to facilitate upright positions during labour [28]. Similarly, research in China found that midwives believed that two midwives were necessary to facilitate upright labour positions during the second stage of labour, a practice often deemed impractical due to staff shortages [27]. Despite these challenges, WHO guidelines state that upright positions do not necessarily require additional equipment or human resources [5]. Hospitals in Uganda face significant staffing shortages, with one midwife overseeing between 350 and 500 deliveries annually—twice the number recommended by the WHO [36]. Furthermore, midwives in Uganda have previously reported a lack of equipment even for the basic provision of maternal care [37], similar to how the midwives in this study described the limited space and the inappropriate environment. These findings imply that it is important to address these challenges with a systemic approach, meaning that healthcare institutions and the government must tackle the broader issue of basic resource shortages and overworked midwives when initiating new labour positions. Moreover, when basic physical and human resources are available, it is crucial to provide training for midwives to utilise these resources effectively.

We found that midwives were driven by their expertise and commitment to maternal and fetal safety, encouraging women to be mobile during the first stage of labour while assuming greater control during the second stage. Other studies have shown that the second stage of labour is typically recognised as an intensive phase for both mother and baby, requiring significant attention from midwives [38]. A study from Tanzania has shown similar findings to this study, where maternal health providers initially encouraged women to be mobile during the first stage but became more directive as labour progressed [6]. The midwives’ reasoning for ensuring the well-being of both mothers and babies aligns with the principles of the midwifery model of care [39]. This model emphasises promoting and safeguarding the health of women and newborns while maintaining trust in women’s inherent abilities during childbirth [39]. However, in this study, at times, the midwives’ perspectives and practices were more closely aligned with the biomedical model, and this could be partly due to inadequate skill or misconceptions, as previously discussed. For instance, they often viewed the lithotomy position as ‘a tradition’ and did not consistently offer women a choice of labour positions. A study in China revealed that midwives indicated that their daily practices were governed by protocols established by the medical model [40]. Additionally, the dominance of the biomedical model in hospital settings might shape midwives’ approaches to care, potentially compromising their adherence to the midwifery model’s principles [41]. Thus, efforts to bridge this gap and promote a more woman-centred approach within medical institutions may be necessary when initiating new labour positions.

Midwives expressed a preference for continuing to adopt prevailing birthing practices, which suggests a tendency to favour established norms. Midwives in other settings have discussed how certain practices are often seen as outside the norm, suggesting the need for courage and determination to fully inform and support women [42]. This inclination towards conformity aligns with Foucault’s concept of normalisation [21, 22], which highlights the pressure to conform to norms within institutional settings like hospitals. Institutional protocols, guidelines, and routines establish a standard way of doing things, shaping the behaviours and practices of professionals, including midwives [21–23]. Additionally, the historical role of the medical system in regulating and controlling the individuals within it could influence midwives’ practice [21, 23]. A meta-synthesis shows that within this institutional culture, managing birth actively in a busy setting, risk-aversion practice, and handling emergencies were prioritised skills [41]. These priorities often took precedence over the provision of woman-centred care or the minimisation of interventions [41]. However, the tendency for midwives in this study to be favourable towards new labour positions that are positively perceived by their peers could also imply the influence of social norms beyond institutional norms. Similar findings were observed in other settings, where midwives acknowledged being influenced by their colleagues more than other healthcare providers or the hospital guidelines [41, 43]. These findings suggest that both institutional and social norms play a role in shaping midwives’ choice of labour position and may influence the provision of care to women during childbirth.

The encouragement of supine birthing positions by midwives in this study may reflect enduring colonial biases against traditional practices within midwifery training. Before colonialism, women in Uganda gave birth with the support of traditional birth attendants and could choose birthing positions that best suited them—including upright positions. However, during the colonial era, nursing and midwifery training in British colonies increasingly focused on exporting British medical ideas and practices “to indigenous populations abroad” [44]. This could include the standardisation of childbirth practices, such as the supine birthing position, which aligned with Western biomedical norms. At the same time, colonial systems often equated traditional medicine with superstition or witchcraft, leading to the suppression of local health practices, including traditional birth practices [45]. Globally, traditional birthing practices have since been cast as backward or harmful within colonial and postcolonial development agendas [46]. The increasing medicalisation of childbirth and the dominance of the supine position in midwifery training may reflect this enduring colonial legacy, where traditional practices continue to be viewed with disfavour and excluded from institutionalised healthcare.

### Study limitations

Within this study, incorporating two separate sets of interviews had both advantages and drawbacks. On the positive side, the information gathered during the first round allowed for the development of an interview guide for the second that was sensitive to the setting and specific hospital context. This made the questions and probes more relevant and tailored to the specific circumstances of the midwives’ work environment. However, since the first round of interviews provided less information, the development of themes relied more heavily on the data from the second round of interviews.

Having multiple interviewers with varying familiarity with the setting can be an advantage and a limitation. Mixing insider and outsider perspectives allowed for a deeper, nuanced understanding, as each highlighted unique aspects of the phenomenon. However, insiders were not inherently more trustworthy, as each interviewer brought their own biases; for example, insiders may have overlooked details they take for granted.

## Conclusion

We found that midwives were open to supporting various labour positions when they felt confident in their safety and efficacy for both mother and infant. When introducing new labour positions into practice, it is essential to address midwives’ need for skills and clarify misconceptions about risks and the need for specialised equipment. The provider-centric norm within healthcare institutions may influence midwives’ care approaches and shape their use of labour positions during childbirth. To facilitate the integration of new labour positions, hospital and policy stakeholders must address the fundamental needs of midwifery care and create an environment that enables midwives to perform their duties confidently.

## Electronic supplementary material

Below is the link to the electronic supplementary material.

Supplementary Material 1

## Data Availability

Data can be accessed upon reasonable request. Relevant information is included in the manuscript and its supporting files. The complete data set cannot be shared publicly due to privacy assurances given to participants and because ethical clearance was granted based on the participants’ anonymity. Excerpts with identifying details removed may be provided to researchers or reviewers who agree to a data-sharing arrangement and adhere to confidentiality protocols. Full transcripts remain accessible only to the researchers involved in the study. Data requests may be directed to the last author, EA (elizabeth.ayebare@mak.ac.ug).

## Abbreviations

ALERT: Action Leveraging Evidence to Reduce Perinatal Mortality and Morbidity
COREQ: Consolidated Criteria for Reporting Qualitative Research
WHO: World Health Organization
