# Supplementary material for: ‘This is like tradition, lie on your back, hold your leg, and push’: understanding midwives’ perspectives on their choice of labour positions in a Ugandan hospital
Source: BMC Pregnancy Childbirth. 2025 May 13;25:564. doi: 10.1186/s12884-025-07657-2 (PMC12070630; doi:10.1186/s12884-025-07657-2)
Supplement: Supplementary file 1 — Supplementary Material 1 [file 12884_2025_7657_MOESM1_ESM.docx]

**Supplementary file 1**

**Interview Questions**

| Subject | Questions |
| --- | --- |
| Sociodemographic information | Tell me about yourself? |
| Labour | 1. When a woman is in labour, what do you usually do? |
| Mobility during the first stage | 1. Can you tell me what position women typically assume during the first stage of labour? |
| Positions during the second stage of labour | 1. What birthing position do the women here typically assume during the second stage of labour? 2. How is the decision to labour position made? 3. Are there any birthing position other than women lying on their back that you have heard or use? |
| Alternative labour positions | 1. Can you tell me about your experience with those birthing positions? 2. Would you be willing to assist the women in these positions? 3. What makes it hard for you to help them to be in those positions? 4. What makes it easy for you to help them to be in those positions? 5. Have you ever used a birthing stool? |
| Final questions | 1. Is there anything that you feel that we have not discussed that you feel was important to be discussed? 2. Do you have any questions you want to ask me? |
